# Oxygen-Glucose Deprivation in Organotypic Hippocampal Cultures Leads to Cytoskeleton Rearrangement and Immune Activation: Link to the Potential Pathomechanism of Ischaemic Stroke

**DOI:** 10.3390/cells12111465

**Published:** 2023-05-24

**Authors:** Natalia Bryniarska-Kubiak, Andrzej Kubiak, Ewa Trojan, Julita Wesołowska, Małgorzata Lekka, Agnieszka Basta-Kaim

**Affiliations:** 1 Laboratory of Immunoendocrinology, Department of Experimental Neuroendocrinology, Maj Institute of Pharmacology, Polish Academy of Sciences, 12 Smętna St., 31-343 Kraków, Poland; 2 Department of Biophysical Microstructures, Institute of Nuclear Physics, Polish Academy of Sciences, 152 Radzikowskiego St., 31-342 Kraków, Poland; 3 Laboratory of Stem Cell Biology, Faculty of Biochemistry, Biophysics and Biotechnology, Jagiellonian University, 7 Gronostajowa St., 30-387 Kraków, Poland; 4 Laboratory for In Vivo and In Vitro Imaging, Maj Institute of Pharmacology, Polish Academy of Sciences, 12 Smętna St., 31-343 Kraków, Poland

**Keywords:** organotypic hippocampal cultures, oxygen-glucose deprivation, cytoskeleton, inflammation, atomic force microscopy

## Abstract

Ischaemic stroke is characterized by a sudden loss of blood circulation to an area of the brain, resulting in a corresponding loss of neurologic function. As a result of this process, neurons in the ischaemic core are deprived of oxygen and trophic substances and are consequently destroyed. Tissue damage in brain ischaemia results from a complex pathophysiological cascade comprising various distinct pathological events. Ischaemia leads to brain damage by stimulating many processes, such as excitotoxicity, oxidative stress, inflammation, acidotoxicity, and apoptosis. Nevertheless, less attention has been given to biophysical factors, including the organization of the cytoskeleton and the mechanical properties of cells. Therefore, in the present study, we sought to evaluate whether the oxygen-glucose deprivation (OGD) procedure, which is a commonly accepted experimental model of ischaemia, could affect cytoskeleton organization and the paracrine immune response. The abovementioned aspects were examined ex vivo in organotypic hippocampal cultures (OHCs) subjected to the OGD procedure. We measured cell death/viability, nitric oxide (NO) release, and hypoxia-inducible factor 1α (HIF-1α) levels. Next, the impact of the OGD procedure on cytoskeletal organization was evaluated using combined confocal fluorescence microscopy (CFM) and atomic force microscopy (AFM). Concurrently, to find whether there is a correlation between biophysical properties and the immune response, we examined the impact of OGD on the levels of crucial ischaemia cytokines (IL-1β, IL-6, IL-18, TNF-α, IL-10, IL-4) and chemokines (CCL3, CCL5, CXCL10) in OHCs and calculated Pearsons’ and Spearman’s rank correlation coefficients. The results of the current study demonstrated that the OGD procedure intensified cell death and nitric oxide release and led to the potentiation of HIF-1α release in OHCs. Moreover, we presented significant disturbances in the organization of the cytoskeleton (actin fibers, microtubular network) and cytoskeleton-associated protein 2 (MAP-2), which is a neuronal marker. Simultaneously, our study provided new evidence that the OGD procedure leads to the stiffening of OHCs and a malfunction in immune homeostasis. A negative linear correlation between tissue stiffness and branched IBA1 positive cells after the OGD procedure suggests the pro-inflammatory polarization of microglia. Moreover, the negative correlation of pro- and positive anti-inflammatory factors with actin fibers density indicates an opposing effect of the immune mediators on the rearrangement of cytoskeleton induced by OGD procedure in OHCs. Our study constitutes a basis for further research and provides a rationale for integrating biomechanical and biochemical methods in studying the pathomechanism of stroke-related brain damage. Furthermore, presented data pointed out the interesting direction of proof-of-concept studies, in which follow-up may establish new targets for brain ischemia therapy.

## 1. Introduction

The versatility of processes leading to neuron death after stroke creates the need for understanding the mechanisms underlying those changes. Hence, the biochemical background involved in neuronal death is currently being intensively investigated; however, less attention has been given to biophysical factors, including the organization of the cytoskeleton and mechanical properties of cells [1,2].

Forces generated by the cellular cytoskeleton and/or the extracellular matrix [3,4] are crucial not only in the process of embryonic development, where they affect cell proliferation and differentiation [5], but are also characteristic of pathological processes, including those related to the brain [6,7,8]. Any aspect of brain cell (neuronal and glial) activity, such as adhesion and/or migration, is mediated by mechanical cues [9]. Moreover, the organization and proper functioning of the cytoskeleton are crucial for the proper transduction of signals from the cell environment into the cell nucleus [10]. Importantly, the mechanical transduction of signals is at least 40 times faster than the transduction of biochemical signals in the cytoplasm [11]; thus, any alteration in cytoskeleton integrity and functionality induces various malfunctions leading to dysregulation of homeostasis. On the other hand, the interplay between the cytoskeleton and physical factors plays a key role in regulating new neuron formation [12]. Seano et al. (2019) found that forces generated by solid stress led to changes in nuclear morphology and subsequent death of neurons in the region affected by the strain generated by the brain tumor mass. Moreover, solid stress generated by the tumor could decrease perfusion in surrounding blood vessels [13]. Similarly, changes in brain stiffness were detected in C57BL/6 mice subjected to middle cerebral artery occlusion (MCAo), probably by changes in fluid distribution caused by brain damage [14]. Considering the physical mechanism of brain damage as a result of the interaction of forces generated in pathological conditions, searching for similar grounds in the case of an ischaemic stroke may be an interesting direction for research. Therefore, we postulate that the OGD procedure ex vivo leads not only to dysregulation of homeostasis and changes in the synthesis of inflammatory factors but may also lead to biomechanical changes in the hippocampal tissue expressed as the cytoskeleton rearrangement.

To verify our hypothesis, we investigated the impact of the OGD procedure on OHCs cytoskeleton organization using combined confocal fluorescence microscopy (CFM) and atomic force microscopy (AFM). CFM was used to determine changes in the organization of crucial cytoskeleton components (actin, tubulin, and MAP-2) under ischemic conditions. Quantitative image analysis was performed in ImageJ (v1.53t) software in order to precisely compare experimental groups. AFM working in force spectroscopy mode was applied to determine the mechanical properties of OHCs.

## 2. Materials and Methods

### 2.1. Animals

Sprague–Dawley rats (200–250 g) were purchased from Charles River (Sulzfeld, Germany) and kept under standard conditions at a temperature of 23 °C with a daily cycle of 12 h of light/12 h of darkness (light from 08:00) and ad libitum access to water and food. After the acclimatization period, the phase of the estrous cycle was determined based on vaginal smears that were obtained daily from the females. On the day of proestrus, the females were placed with males for 12 h, and the presence of sperm in vaginal smears was checked the next morning. All experimental protocols were performed in accordance with guidelines from the Committee for Laboratory Animal Welfare and Ethics of the Maj Institute of Pharmacology, Polish Academy of Sciences, Krakow, Poland. All possible efforts were made to minimize the number of animals used and their suffering.

### 2.2. Organotypic Hippocampal Cultures (OHCs)

Organotypic cultures were established from the hippocampus of P6-7 female rats according to the method described by Stoppini et al. (1991) with slight modifications [15,16]. Before isolating the brain, P6-7 rats were placed on ice and then decapitated. The dissected brains were immediately placed into an ice-cold working buffer containing 96% Hank’s balanced salt solution (HBSS, 1 M; Gibco, Waltham, MA, USA), 3.5% glucose (Sigma–Aldrich, St. Louis, MO, USA), and 0.5% penicillin and streptomycin (Gibco, Waltham, MA, USA). The hippocampus was isolated and then transferred to Teflon disks and cut into 350 µm thick sections using a tissue chopper (McIlwain, TedPella, Redding, CA, USA). The cut sections were rinsed from the Teflon disc into the working buffer, and after initial optical microscopy analysis, they were transferred to ThinCert™ membranes (Greiner Bio-One, Austria) and placed in 6-well plates (Greiner Bio-One, Kremsmünster, Austria). Five slices of the hippocampus were placed on each ThinCert™ membrane. Each well was filled with 1 mL of 50% DMEM + GlutaMax™-I medium (Gibco, Waltham, MA, USA) containing 20% HBSS, 25% horse serum, 5 mg/mL glucose, 2% B-27 supplement, 2% fungizin, penicillin at a concentration of 100 U/mL, and streptomycin at a concentration of 0.1 mg/mL. OHCs were carried out in an incubator at 37 °C in an atmosphere containing 95% air and 5% CO_2_. The culture medium was changed every other day, gradually reducing the concentration of horse serum (from Day 4 to Day 7). On the 7th day of cultivation, the medium was serum-free and contained 50% DMEM F-12, 20% HBSS, 5 mg/mL glucose, 2% B-27 supplement, 2% N2 supplement, 2% fungizin, 100 U/mL penicillin, and 0.1 mg/mL streptomycin.

### 2.3. Oxygen-Glucose Deprivation (OGD)

**Figure 1:**
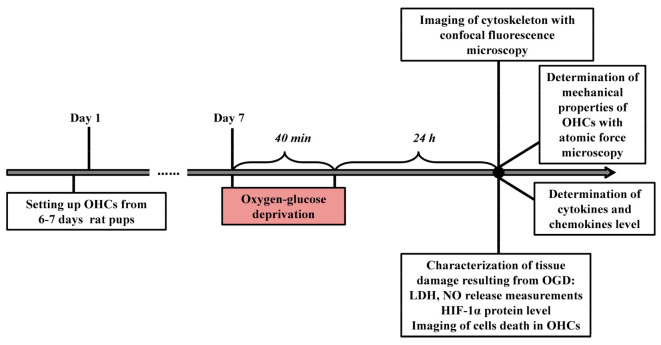
Schematic of the experimental setup (OHCs—organotypic hippocampal cultures, LDH—lactate dehydrogenase, NO—nitric oxide, HIF-1α—hypoxia-inducible factor 1α).

The OGD procedure was performed on the 7th day, after the establishment of the culture according to the protocol of Sarnowska et al. (2009) with slight modifications [17]. Hippocampal cultures were washed twice in Ringer’s solution containing 10 mM mannitol (Sigma–Aldrich, St. Louis, MO, USA). Then, the membranes were transferred to new 6-well plates containing 1 mL Ringer’s solution and placed in a hypoxic chamber (37 °C; gas flow: 95% N_2_, 5% CO_2_) for 40 min. Next, inserts with hippocampal slices were transferred back to serum-free plates and grown under standard conditions (37 °C; 5% CO_2_). The actual experiments were carried out for 24 h after the OGD procedure (Figure 1).

### 2.4. Propidium Iodide Staining

Due to its chemical properties, propidium iodide (PI) (Sigma–Aldrich, St. Louis, MO, USA) is routinely used to determine cell mortality. PI does not cross the cell membrane continuously but stains DNA released from the cells, of which the membranes are perforated and emit orange-red light when excited with blue light; therefore, it is used for fluorescence labelling of dead cells [18]. In the conducted research, 24 h after the OGD procedure, PI was added to the culture medium at a concentration of 2 μM [19], and then, after 2 h of incubation, its capture was visualized using a fluorescence microscope (Ex/Em: 536/620 AxionCam MRm, ZEISS, Jena, Germany).

### 2.5. LDH Assay

When cells are damaged (disruption of the cell membrane), the enzyme lactate dehydrogenase (LDH) is released into the culture medium. In the test used, one of the components of the reaction mixture was lactic acid, which is converted by LDH to pyruvic acid, with the simultaneous reduction of NAD+ to NADH. In the second stage of the reaction, the enzyme (diaphorase) oxidizes NADH and reduces the substrate to a colored product (formazan). The assessed color intensity is proportional to the cytotoxicity of the tested compounds/chemicals. The LDH test was carried out following the method described by Ślusarczyk et al. [20]. Twenty-four hours after the OGD procedure, 50 μL of the medium was taken, and 50 μL of the reagent mixture (consisting of the catalyst and the dye solution) provided by the manufacturer of the kit (Cytotoxicity Detection Kit, Roche, Mannheim, Germany) was added, followed by 15 min of incubation at room temperature. The absorbance (wavelength λ = 490 nm) was measured with an Infinite^®^ M200 PRO automatic microplate reader (TECAN, Männedorf, Switzerland). The data were normalized to the activity of LDH released from control, vehicle-treated slices (100%) and are expressed as a percentage of the control ± SEM (standard error of the mean).

### 2.6. NO Release Assay (Nitrite Ion in Solution)

Nitric oxide (NO) secretion by OHCs was assessed using the Griess reaction, in which the extracellular release of nitrite (NO^2−^) was measured as previously described [16,21]. Twenty-four hours after the OGD procedure, 50 μL of the medium was mixed in a 1:1 ratio with a mixture of the following reagents: Griess A (0.1% N-1-naphthyl ethylenediamine dihydrochloride) and Griess B (1% sulfanilamide in 5% phosphoric acid) in a 96-well plate and incubated for 10 min at room temperature. The absorbance at λ = 540 nm was then measured using an Infinite^®^ M200 PRO automatic microplate reader (TECAN, Männedorf, Switzerland). The data were normalized to NO released from control, vehicle-treated slices (100%) and are expressed as a percentage of the control ± SEM.

### 2.7. Enzyme-Linked Immunosorbent Assay (ELISA)

Twenty-four hours after the OGD procedure, OHCs medium was collected to assess IL-6, TNF-α, IL-10 and IL-4 levels. The protein levels of cytokines, including interleukin 6 (IL-6; obtained from Bioassay Technology Laboratory, Birmingham, UK), tumor necrosis factor α (TNF-α;obtained from Bioassay Technology Laboratory, Birmingham, UK), interleukin 10 (IL-10; obtained from Bioassay Technology Laboratory, Birmingham, UK and interleukin 4 (IL-4; obtained from Bioassay Technology Laboratory, Birmingham, UK), were measured in the culture medium using commercially available kits according to the manufacturer’s instructions.

Additionally, 24 h after the OGD procedure, the OHCs were sonicated in RIPA buffer containing 1 mM sodium orthovanadate, 1 mM PMSF, 10 µg/mL protease inhibitors (P8340), and 10 µg/mL phosphatase inhibitors (P2850; P5726) (all obtained from Sigma–Aldrich, St. Louis, MO, USA). Then, the homogenates were shaken on ice for 30 min and centrifuged for 20 min at 4 °C at a speed of 14,000 rpm. The protein concentrations of the analyzed samples were determined using a BCA Protein Assay Kit (Sigma-Aldrich, St. Louis, MO, United States) and measured at a wavelength of 562 nm using a Tecan Infinite 200 Pro spectrophotometer (TECAN, Männedorf, Switzerland) in triplicate for each sample. Moreover, an ELISA kit for the HIF-1α factor (Rat Hypoxia-Inducible Factor 1α; obtained from Cusabio, Houston, TX, USA) was used according to the manufacturer’s instructions. The detection limits were as follows: TNF-α < 5 pg/mL, IL-6 < 0.1 pg/mL IL-10 < 3 pg/mL, IL-4 < 1 pg/mL, and HIF-1α < 3.12 pg/mL. The inter-assay precision of all ELISA kits was CV% < 10%. The intra-assay precision of all ELISA kits was CV% < 8%.

### 2.8. MILLIPLEX^®^ Assay

Using the Milliplex Map Rat Cytokine/Chemokine Magnetic Bead Panel (Merck-Millipore, Darmstadt, Germany) method in an OHCs medium, the levels of the cytokines IL-1β (interleukin 1β), IL-18 (interleukin 18) and the chemokines CCL3 (chemokine (C-C motif) ligand 3; MIP-1α), CCL5 (chemokine (C-C motif) ligand 5; RANTES), and CXCL10 (C-X-C motif chemokine ligand 10; IP-10) were assessed 24 h after the OGD procedure. The samples were applied to 96-well plates in volumes according to the manufacturer’s instructions. The measurement was made with a MAGPIX^®^ automatic reader (Luminex Corporation, Northbrook, IL, USA), and the analysis was performed with xPONENT^®^ (v3.1) software (Luminex Corporation, Northbrook, IL, USA).

### 2.9. Immunofluorescence Staining

Immunofluorescent staining of OHCs was performed according to a protocol established by Gogolla et al. [22]. Immunofluorescence staining of OHCs was performed after the OGD procedure. The cultures were fixed in 4% paraformaldehyde, rinsed three times with phosphate-buffered saline (PBS), and treated with 20% methanol. After another wash with PBS, the cultures were permeabilized with 0.05% Triton solution, and nonspecific binding was blocked with 20% bovine serum albumin (BSA, Sigma-Aldrich, St. Louis, MO, USA) overnight (at 4 °C). Next, the cells were incubated with the appropriate inline antibody (Table 1) overnight at 4 °C. Following washing with PBS, the cultures were incubated for 4 h at room temperature with the appropriate secondary antibody (Table 2). At the end of the procedure, labelling with DAPI or Hoechst 33,342 dyes was performed to visualize the cell nuclei. The preparations were visualized on a Leica TCS SP8 X confocal fluorescence microscope (Leica Microsystems CMS GmbH, Mannheim, Germany).

### 2.10. Confocal Fluorescence Imaging

The photos were taken using a Leica TCS SP8 X confocal laser scanning microscope (Leica Microsystems CMS GmbH, Mannheim, Germany) using a 10× objective and an immersion objective with 63× magnification (HC PL APO CS2 1.40 NA OIL). Image acquisition was performed bidirectionally along the X-axis with a scanning frequency of 200 Hz, an image format of 1648 × 1648 (pixel size 74 nm), and a digital magnification of 1.5; to reduce noise, images were recorded with a line average of 3. Analysis was performed using ImageJ v1.53t software (Wayne Rasband, National Institute of Health, Bethesda, MD, USA) with default parameters.

### 2.11. Determination of Young’s Modulus Using Atomic Force Microscopy

**Figure 2:**
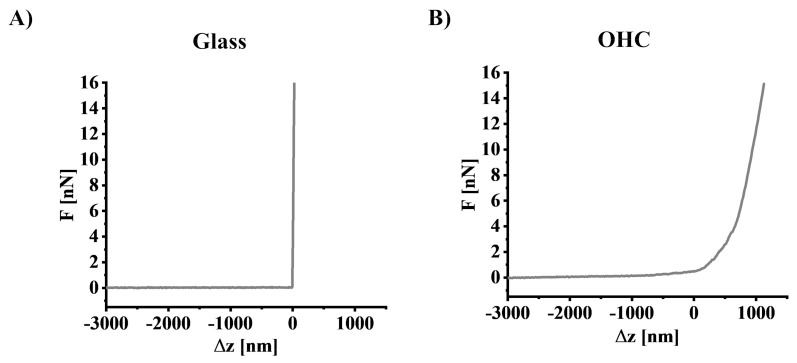
Examples of force-distance curves recorded: (**A**) on a non-deformable substrate, which here was a glass coverslip, and (**B**) on OHC.

Measurements of the mechanical properties of OHCs were carried out using atomic force microscopy (AFM, model XE-120, Park Systems, South Korea) operating in force spectroscopy mode. Silicon nitride cantilevers (NSC36-C, MikroMasch, Sofia, Bulgaria) with nominal spring constant k = 0.6 N/m and open angle of α = 40° were used for the measurements. Before the measurements, the spring constant was calibrated using the thermal noise method [23], while the sensitivity of the photodetector was determined by recording the force-distance curve on a non-deformable substrate (in this case, a glass coverslip, Figure 2A).

AFM measurements were conducted on fixed samples according to Targosz-Korecka et al. (2015) protocol with modifications [24]. OHCs were fixed by 60 min of incubation in a 4% paraformaldehyde solution. Subsequently, OHCs were cut from the rim of the membrane and glued to a glass coverslip by applying a drop of cyanoacrylate to the rim of the membrane (to avoid contamination of the tissue). For the measurement, the coverslips were placed in a liquid cell setup filled with PBS. Force spectroscopy was performed within a 4-pixel × 4-pixel grid spanning the area from 10 µm × 10 µm to 26.7 µm × 26.7 µm in the CA1 region of the hippocampus. The maximum load force of 15 nN was applied during spectroscopic measurements, while the surface topography was recorded with a set point force of 1 nN. The obtained force-distance curves (Figure 2B) were converted to the force-indentation curves and used to calculate Young’s modulus based on the Hertz-Sneddon contact mechanics assuming that the shape of the probing tip can be approximated by a cone [25].

The following equation was applied to obtain Young’s modulus:

$$F\left(\Delta z\right)=\frac{2\cdot tan\theta }{\pi }\cdot \frac{E}{1-\mu^{2}}\Delta z^{2}\;$$

where *F* is the load force, Δ*z* is the indentation, α is the open angle of the probing cone, *E* is Young’s modulus, and *µ* is the Poisson’s ratio of the studied sample (assumed to be 0.5, indicating incompressible material).

### 2.12. Quantitative Analysis of Fluorescent Images

Images were analyzed according to the protocol described in our previous work [21] using Fiji ImageJ (v1.53t) software. Briefly, all images which were recorded in order for quantitative data analysis were acquired with fixed imaging parameters. A proper threshold was applied to determine the cell spread area and then a particle analysis tool was used. Intensity and counting of cells were obtained with basic Fiji ImageJ functionalities. 

### 2.13. Statistical Analysis

All statistical analyses were performed using GraphPad Prism 5. To identify the extreme values, the Grubbs test was used. The normality of the distributions was assessed by the Shapiro–Wilk test. The significance of differences between the compared groups was assessed using Student’s t-test or the Mann–Whitney U test. Information about the number of OHCs analyzed in each experiment is provided in figure legend-each dot presents data acquired from one analyzed sample. Each sample was one well with insert on which five hippocampal slices were cultured. The results obtained in the ELISA/MILLIPLEX^®^ methods were presented as pg/mg of protein and ng or pg/mL of the culture medium, while the results obtained from the determinations using the atomic force microscope were expressed as kPa (median values of Young’s modulus ± median absolute deviation). Due to the asymmetric nature of the distributions of Young’s modulus values, the medians and the nonparametric test were used to evaluate the statistical significance of the studied groups. The following values were considered statistically significant: *p < 0.05, ** *p* < 0.01, *** *p* < 0.001. 

To find whether there is a correlation among various parameters obtained in our study, we calculated Pearsons’ and Spearman’s rank correlation coefficients. The former describes a linear correlation between two variables, while the latter indicates the non-parametric monotonicity of the relation between two variables. In both cases, the coefficients close to 1 (or −1) denotes a perfect association of two corresponding variables (positive or negative, respectively). 

## 3. Results

### 3.1. The Impact of Oxygen-Glucose Deprivation on the OHCs Damage 

**Figure 3:**
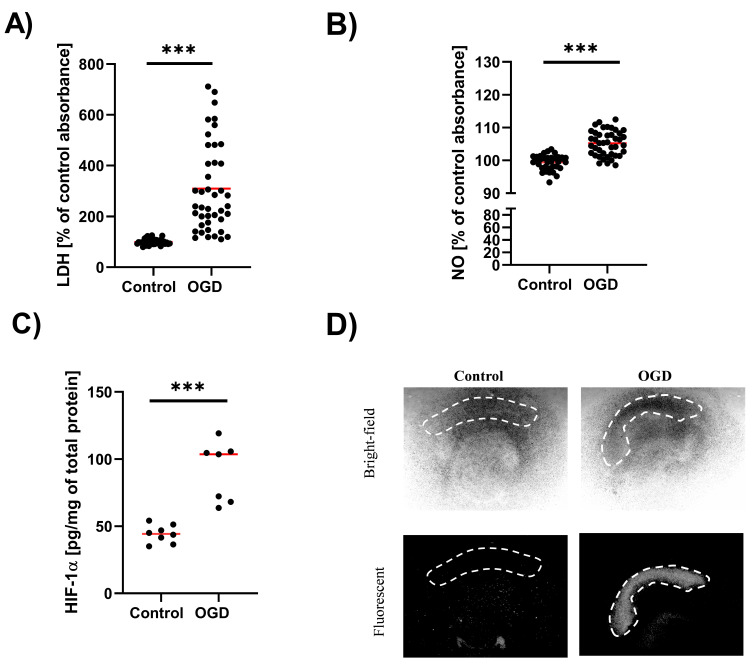
Validation of the OGD procedure in OHCs. (**A**) The level of LDH release in OHC samples after OGD. The results are presented as a percentage of the control. Each dot denotes LDH release measurement from one OHC sample. (**B**) The level of NO released from OHC samples after OGD The results are presented as a percentage of the control. Each dot denotes NO release measurement from one OHC sample. (**C**) The level of HIF-1α in OHC samples after OGD. The results are expressed as pg/mg protein. Each dot denotes the protein level at one OHC sample. (**D**) Representative microscopic photos of OHCs samples. Pictures were taken at 2.5× magnification in a bright field and using a fluorescence microscope. Control and OGD cultures were stained with propidium iodide (PI). The dashed white line indicates the CA1 region of the hippocampus. *** *p* < 0.001.

In the first stage of the study, we assessed the effect of OGD on the release of LDH. This test takes advantage of the fact that along with cell damage (disruption of the cell membrane), the cytoplasmic enzyme LDH is released into the culture medium. The results showed that the OGD procedure increased LDH release (*p* < 0.001) (Figure 3A). Moreover, in OGD-treated samples a linear correlation was found between tissue stiffness and LDH level (Pearson’s coefficient = 0.30782).

Next, we used a method based on the Griess reaction to assess the impact of OGD on NO secretion. In this case, we observed that the OGD procedure led to a significant increase in NO secretion (*p* < 0.001) (Figure 3B).

HIF-1α is a transcription factor that is degraded when the oxygen concentration is higher than 5%. When the oxygen concentration drops below this level, HIF-1α is maintained stably in cells and plays versatile roles as a transcription factor modulating multiple pathways [26,27]. Therefore, in the next set of experiments, the level of HIF-1α protein in OHCs subjected to OGD was examined. Cultures under OGD conditions had significantly increased levels of HIF-1α compared to control cultures (*p* = 0.0011) (Figure 3C).

Finally, we aimed to visualize the area within OHCs affected by OGD. Thus, we performed fluorescent imaging of OHCs stained with PI. In control cultures and 24 h after the OGD procedure, the uptake of PI was assessed using bright-field microscopy and fluorescence microscopy. Imaging showed that OGD damaged OHCs, especially in the hippocampal CA1 region (Figure 3D).

### 3.2. The Impact of Oxygen-Glucose Deprivation on Neuronal and Microglial Cells in OHCs

**Figure 4:**
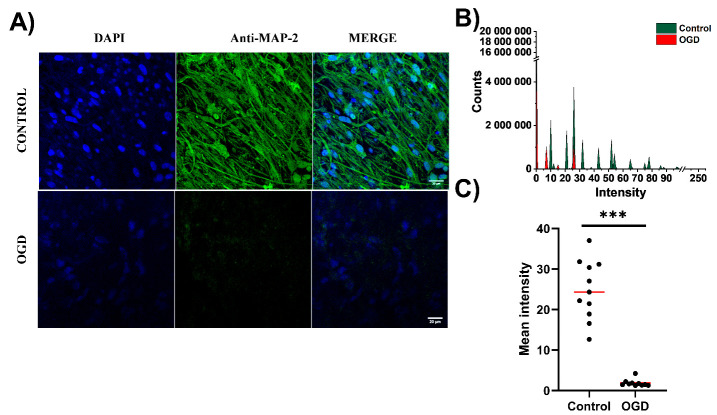
MAP-2 imaging in OHCs subjected to OGD. (**A**) Representative pictures of the CA1 area of the hippocampus stained with anti-MAP-2 antibody (neuron marker) and DAPI (nuclei). The photos were taken using a confocal microscope. Magnification ×63, scale 20 µm. (**B**) Area plots showing the distribution of all intensity values for MAP-2 for control (green) and OGD-subjected (red) OHCs. (**C**) The mean intensity for MAP-2 staining for control and OGD-treated OHCs. Each dot presents the mean intensity from one analyzed image, red line denotes the median value. All comparisons between groups were conducted with the use of the Mann U Whitney test. *** *p* < 0.001.

In the next set of experiments, we evaluated the impact of the OGD procedure using fluorescence imaging of OHCs stained for the neuronal marker MAP-2. Imaging of whole OHCs showed decreased expression of MAP-2 in OHCs subjected to OGD (Figure 4A). As bright-field and fluorescence microscopy staining of OHCs with PI showed the greatest damage in the CA1 region (Figure 3D), we carried out further visualization focused on this hippocampus region. In control OHCs, MAP-2 expression was very pronounced, with a relatively uniform MAP-2 distribution, including both soma and dendrites. Cell nuclei were visible on different planes, proving the three-dimensional organization of cells within OHCs. Conversely, in OHCs undergoing OGD procedures, MAP-2 abundance was robustly decreased. Using Fiji ImageJ, we quantified fluorescent intensity for MAP-2 staining of investigated images. We observed a significant shift of intensity values toward lower values for OHCs undergoing OGD (Figure 4B). Consequently, while comparing mean values derived from fluorescent intensity histograms for each analyzed image we observed that the median of those values is significantly lower for OGD (1.57) than for control OHCs (24.35), *p* < 0.0001 (Figure 4C). This observation proved that OGD affects the population of neuronal cells in OHCs (Figure 4).

**Figure 5:**
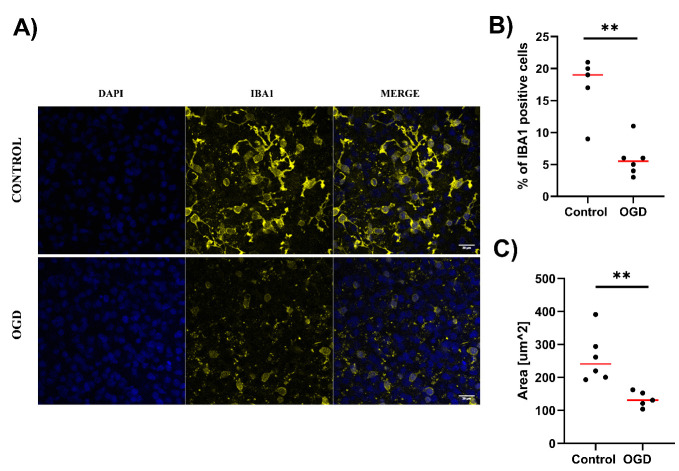
Imaging of the microglial marker IBA1 in OHCs subjected to OGD. (**A**) Representative pictures of the CA1 area of the hippocampus stained with anti-IBA1 antibody (neuron marker) and DAPI (nuclei). The photos were taken using a confocal microscope. Magnification ×63, scale 20 µm. (**B**) The percentage of IBA1-positive cells in images of the CA1 region of the hippocampus. Each dot presents data determined from one analyzed image, red line denotes the median value. (**C**) The cell spread area of IBA1-positive cells in images of the CA1 region of the hippocampus. In control OHCs, 184–229 cells were analyzed per image, while 183–219 cells were analyzed per image in OGD-subjected OHCs. Each dot presents data determined from one analyzed image. Red line denotes the median value. All comparisons between groups were conducted using the Mann U Whitney test. ** *p* < 0.01.

We also aimed to assess the effect of the OGD procedure on microglial cells, which play a crucial role in the inflammatory response in the brain [28,29]. Therefore, we imaged OHCs stained for IBA1 (Figure 5A). Microglial cells were abundant in CA1 in control OHCs (Figure 5A,B). We observed that the median percentage of IBA1 positive cells in CA1 images of control OHCs was 19% (Figure 5B). They possessed branched cytoplasmic extensions and a median cell spread area of 240.8 µm^2^ (Figure 5A,C). After OGD, a significantly (*p* = 0.0065) lower percentage of IBA1-positive microglial cells (median 5.5%) was observed in images of the CA1 region within OHCs (Figure 5B). IBA1-positive cells under OGD conditions were characterized by a lack of branched extensions and significantly decreased cell spread area (131.5 µm^2^, *p* = 0.0043; Figure 5A,C). In OGD-treated samples, a negative linear correlation was found between tissue stiffness and branched IBA1 positive cells (Pearson’s coefficient = −0.981), suggesting the pro-inflammatory polarization of microglia. 

### 3.3. Impact of Oxygen-Glucose Deprivation on the Architecture of the Microtubular and Actin Cytoskeleton of OHCs

**Figure 6:**
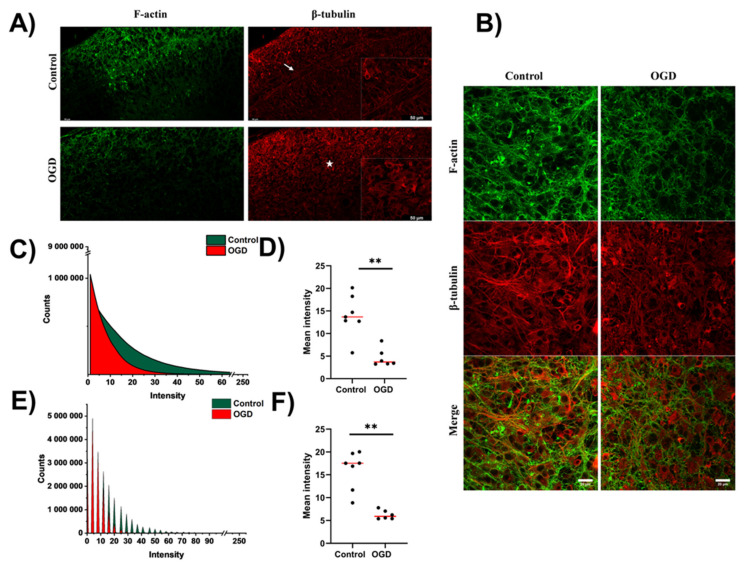
Imaging of actin and the microtubular cytoskeleton in OHCs undergoing OGD. (**A**) Panoramic images of the CA1 area of the hippocampus were obtained with a confocal fluorescence microscope. Actin staining was performed with phalloidin conjugated with Alexa Fluor 488 dye, and microtubules were stained with anti-β-tubulin conjugated with Cy3 dye. Magnification ×4, scale 50 µm. (**B**) Images of the CA1 area of the hippocampus were obtained with a confocal fluorescence microscope. Actin staining was performed with phalloidin compressed with Alexa Fluor 488 dye and microtubules with anti-β-tubulin antibody conjugated with Cy3 dye. Magnification ×63, scale 20 µm. (**C**,**E**) Area plots showing the distribution of all values of intensity for (**C**) actin and (**E**) β-tubulin for control (green) and OGD-subjected (red) OHCs. (**D,F**) Impact of OGD on mean intensity for (**D**) actin and (**F**) β-tubulin staining. Each dot presents the mean intensity from one analyzed image, and the red line denotes the median value. All comparisons between groups were conducted using the Mann U Whitney test. ** *p* < 0.01.

Here, we aimed to visualize the organization of the actin and β-tubulin cytoskeleton in cells composing OHCs. To achieve this goal, we stained OHCs with Alexa 488-conjugated phalloidin, which binds to actin, and Cy3-conjugated anti-β-tubulin antibody. First, we performed panoramic imaging of the CA1 region of the hippocampus to determine whether OGD affects the layered structure of hippocampal tissue. Panoramic imaging of the CA1 region of the hippocampus showed that in the control cultures, both the actin and microtubular cytoskeleton reflected the layered structure of the culture (Figure 6A, white arrow). In contrast, the OGD procedure obliterates the layered organization within the CA1 region of the hippocampus (Figure 6A, white asterisk). Moreover, panoramic imaging of samples revealed that the actin cytoskeleton was generally multibranched in control cells, while it tended to form “sponge-like” structures in OHCs after the OGD procedure (Figure 6A).

Imaging of the CA1 region of the hippocampus performed with higher resolution showed that the control OHCs were characterized by a well-organized actin cytoskeleton and microtubular network (Figure 6B), while the OGD procedure affected the organization of the actin cytoskeleton towards a more diffuse and “spongy state” (Figure 6B). Quantitative observations confirmed those qualitative observations. The distribution of intensity values for Alexa488 was shifted toward lower values in OGD-subjected OHCs (Figure 6C). We also compared mean values derived from fluorescent intensity histograms for each analyzed image and observed that the median of those values is significantly lower for OGD (3.68) than for control OHCs (13.68), *p* = 0.0023 (Figure 4D). 

To obtain a comprehensive view of cytoskeleton organization in OHCs, we also have quantified images showing the microtubular cytoskeleton within OHCs. Similarly, as in the case of the actin cytoskeleton, we observed a shift toward lower fluorescence intensity values in OGD-subjected OHCs compared to the control (Figure 6E). Consequently, the median of the mean intensity value was significantly (*p* = 0.0012) higher for control (17.52) than OGD subjected (5.89) OHCs (Figure 6F). Interestingly, significance of the difference in mean intensity values was lower in the case of microtubules (Figure 6E, *p* = 0.0012) than MAP-2 (Figure 4C, *p* < 0.0001), indicating that microtubules, although malformed after OGD, resist it more than associated with microtubules neuronal marker MAP-2.

### 3.4. The Impact of Oxygen-Glucose Deprivation on the Mechanical Properties of OHCs 

**Figure 7:**
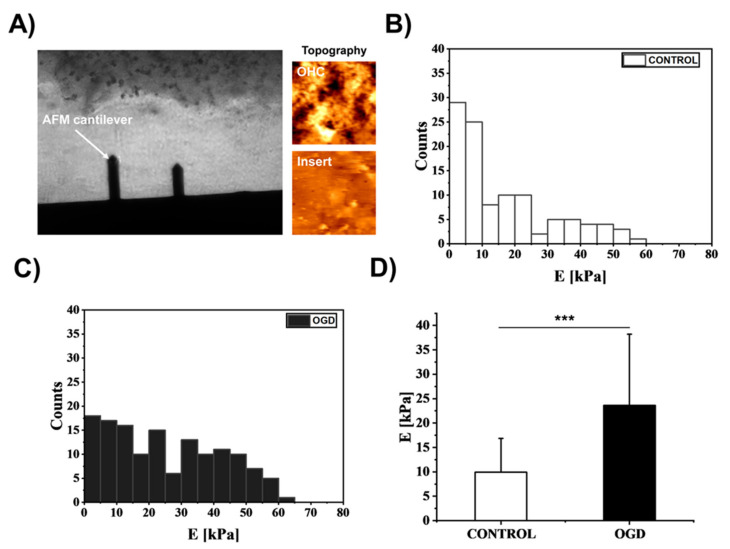
Characterization of the mechanical properties of OHCs exposed to OGD. (**A**) Diagram of the experimental setup used to measure the mechanical properties of OHCs. The optical microscope image shows the AFM cantilever above the sample and the tissue-insert boundary (topography images are shown accordingly). (**B**,**C**) The distribution of Young’s modulus values for control CA1 (**B**) and OGD CA1 (**C**). The bin size was set to 5 kPa. (**D**) The median comparison between control and OGD-treated OHCs. The columns represent the median value of the elastic modulus, and the error bars are the median absolute deviation. The median was obtained from a distribution of at least 30 maps acquired over CA1 regions per condition. *** *p* < 0.001.

The observed changes in the organization of both the microtubular and actin cytoskeletons suggest that the OGD procedure can affect the mechanical properties of OHCs. To verify this hypothesis, AFM was used in force spectroscopy mode. Figure 7A shows an exemplary microscopic image of the measurement, during which the exemplary topographies of the OHC surface and insert were measured comparatively (Figure 7A). The topography images show a heterogeneous and folded OHC structure and a homogeneous and smooth insert topography.

The analysis of Young’s modulus values obtained from the AFM measurements showed that in the case of the control cultures, a larger pool of Young’s modulus with a low modulus of elasticity was obtained (i.e., in the range from 0 to 10 kPa) (Figure 7B), with a simultaneous reduction in the number of modules with higher values, which additionally decreased as the stiffness of the tested samples increased (Figure 7B). This observation suggests that the control cultures show a relatively higher susceptibility to deformation, while after the OGD procedure, the number of modules with low values was significantly reduced and, at the same time, indicated an increase in the number of modules with higher values (Figure 7C). Therefore, it can be postulated that the OGD procedure significantly modulates OHCs stiffness and leads to increased heterogeneity in the mechanical properties of cultures. Due to the asymmetric nature of the distributions of Young’s modulus values, the medians and the nonparametric test were used to evaluate the statistical significance of the studied groups. The median elastic modulus of OHCs increased from 9.95 ± 6.89 kPa in the control condition to 23.63 ± 14.56 kPa after OGD (*p* < 0.001) (Figure 7D).

### 3.5. The Impact of Oxygen-Glucose Deprivation on the Levels of Pro- and Anti-Inflammatory Factors in OHCs

The observed biomechanical changes in OHCs are crucial in the context of recent data showing that the mechanical properties of cells and tissues may determine the profile of the immune response. Therefore, in the next set of experiments, we measured the OGD-induced protein levels of pro- and anti-inflammatory factors, namely, cytokines and chemokines in OHCs supernatants.

**Figure 8:**
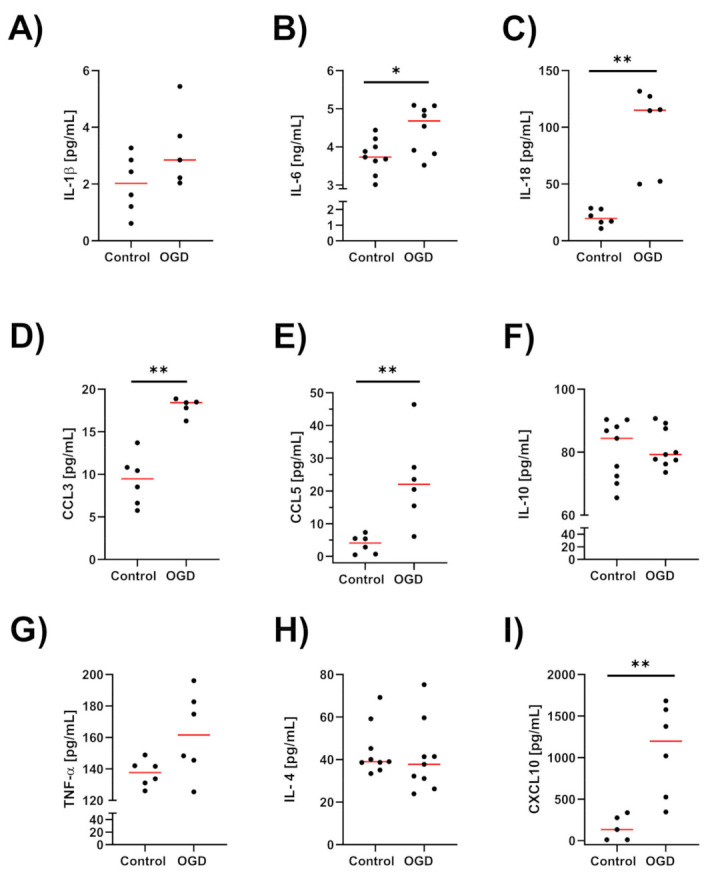
The levels of the pro-inflammatory factors (**A**) IL-1β, (**B**) IL-6, (**C**) IL-18, (**G**) TNF-α, (**D**) CCL3, (**E**) CCL5, and (**I**) CXCL10 and the anti-inflammatory factors: (**H**) IL-4 and (**F**) IL-10 in OHCs. Each dot denotes the factors release measurement from one OHC, red line denotes median value * *p* < 0.05, ** *p* < 0.01. TNF-α—tumor necrosis factor α; IL—interleukin. * *p* < 0.05, ** *p* < 0.01.

As shown in Figure 8, the analyses of OHCs samples revealed that OGD significantly increased the levels of the pro-inflammatory cytokines IL-6 (*p* = 0.036), IL-18 (*p* = 0.0022) and the chemokines CCL3 (*p* = 0.0043), CCL5 (*p* = 0.0043), and CXCL10 (*p* = 0.0043). In the case of IL-1β and TNF-α, an upwards trend in their level was observed after the OGD procedure (*p* = 0.07652) and (*p* = 0.13) respectively. Among the tested markers of the anti-inflammatory phenotype, the level of IL-10 and IL-4 did not change significantly. Taken together, these observations proved that OGD induces a strong inflammatory response in OHCs. 

Interestingly, Pearson’s and Spearman’s rank among parameters demonstrated a correlation between pro- and anti-inflammatory factors, indicating the complex and mutual picture of the inflammatory response in OGD-subjected OHCs including: IL-6 and CCL3 (Pearson’s coefficient = 0.90893, IL-6 and IL-10 (Pearson’s coefficient = 0.72331), and IL-6 and IL-4 (Pearson’s coefficient = −0.76087). Furthermore, in OGD-subjected OHCs, a strong negative linear correlation between CCL3 and actin mean density (Pearson’s coefficient = −0.98955), while positive between IL-4 and actin mean density (Pearson’s coefficient = 0.8743457), was found. It can indicate an opposing effect of pro- and anti-inflammatory factors on the organization of the cytoskeleton in the course of the immune response induced by OGD. 

## 4. Discussion

The role of the cytoskeleton and biophysical properties are currently intensively explored in various physiological and pathological processes including those observed in the central nervous system. Nevertheless, little is known about the significance of cytoskeleton rearrangements in the course of neuroinflammation, which is one of the hallmarks of ischemia. 

In our research, OHCs were used to study the cytoskeleton organization, physical cues, and neuroimmune basis of ischaemic brain damage induced by the OGD procedure. The main advantage of the OHCs model is the ability to replicate many aspects of the in vivo context. OHCs largely retain the studied tissue structure and not only the functional axis between neuron-glia cells but also interactions between the nervous, endocrine, and immune mediators [30]. Most frequently, OHCs obtained from 6- to 7-day-old pups are used because, in this period, the brain has a high degree of plasticity and is resistant to mechanical injuries [31]. Due to these advantages, OHCs are widely used to study the molecular mechanisms of brain diseases, such as depression [32], Alzheimer’s disease [33], and Parkinson’s disease [34]. In the present study, we used the OGD procedure as a model of ischaemic stroke [35], since it can mimic the pathophysiologic events after ischemia in vivo, including selective cell damage and the inflammatory response. OGD duration is critical for damage occurring within OHCs. In fact, increasing OGD duration from 30 to 60 min led to a two-fold increase in Caspase 3 activity and intensity of PI staining of organotypic spinal cord cultures [36].Thus in our research, we decided to set up OGD time for an intermediate time of 40 min according to already published work by Figiel-Dąbrowska et al. [37].Importantly, we observed a similar distribution of dead cells in PI staining (Figure 3D) as observed by other authors for 40 min OGD. The induced injuries directly affect cell morphology, which suggests changes in the mechanical properties of treated cells in response to OGD [16,38,39,40]. Recently, our study showed that the OGD procedure enhanced LDH release and NO production in OHCs. LDH is a stable cytoplasmic enzyme that is rapidly released into the cell culture medium when cell membrane damage occurs [41]. Moreover, imaging with the use of fluorescence microscopy showed that the greatest damage, expressed as PI uptake, affected the CA1 area of the hippocampus. Although PI staining does not clearly distinguish the type of cell death (apoptotic from necrotic), it correlates well with the total number of dead cells [42,43]. In addition, we found that the OGD procedure potentiated the release of NO, which is mainly produced in the brain after the induction of iNOS expression in glial cells [21,44]. Generally, it is a potentially neurotoxic factor because excessive production of NO results in the formation of peroxynitrite by reacting with superoxide, leading to disturbances in mitochondrial processes [45]. Nevertheless, the physiological significance of OGD-induced NO release remains uncertain because of the ability of NO to promote neuronal survival or death, depending on the NO concentration and the site of action [46].

As a follow-up, we found that the OGD procedure caused an increase in the protein level of HIF-1α, which is a subunit of the heterodimeric transcription factor HIF-1. HIF-1α protein is degraded in an environment when the oxygen concentration is above 5%. When the oxygen concentration drops below this value, the HIF-1α and HIF-1β subunits form stable heterodimers showing transcriptional activity and modulating the expression of various genes [27,47]. Mouse neurons exposed to OGD show an increased level of DNA bonded by HIF-1 [48]. Nevertheless, in an in vitro OGD ischaemia model, deletion of the HIF-1 gene in neurons increased their mortality, while a stabilizer of HIF-1 heterodimers (DMOG compound) reduced cell death in this model [49]. In contrast, in in vivo studies (a mouse model of neonatal ischaemia), an inverse relationship was observed, since the HIF-1-2-methoxy oestradiol (2-methoxy oestradiol—2ME2) inhibitor exhibited a neuroprotective effect and reduced brain oedema as well as blood–brain-bridge permeability [50]. These controversial results show the importance of selecting an experimental model and clearly suggest the need for further research on this issue.

Confocal microscopy was employed to visualize the impact of the OGD model on glial and neuronal cells, especially in the CA1 region, where the greatest damage after OGD was found (Figure 4, Figure 5 and Figure 6). Thanks to the use of Fiji ImageJ, we could perform quantitative analysis of acquired data, including the determination of fluorescent intensity [51]. This method allowed for assessing in a quantitative way the expression of MAP-2, as well as a disturbance in cytoskeleton lattice solidity for both actin and microtubular cytoskeleton. Since our goal was to assess microglia morphology, we quantified the spread area of microglia cells as described in our previous work [21]. It is well known that microglia play a crucial role in the inflammatory response in the brain, but their prolonged and increased pro-inflammatory activation is also important in subsequent neuronal damage. In our study, we imaged OHCs stained for IBA1. In control OHCs, we observed that microglial cells were abundant in the CA1 region and possessed branched cytoplasmic extensions. However, after the OGD procedure, a visibly lower number of microglial cells was observed. Nevertheless, IBA1-positive cells under OGD conditions were characterized by a lack of branched extensions and smaller circular somas. Although these observations have some limitations, they may indicate significant morphological changes in microglia induced by the OGD procedure, which is in line with the observations of other authors in slightly different experimental models [52]. However, it must be noted that recent data strongly emphasize the “mixed” phenotype of microglial activation during ischaemia and/or a shift in the profile of microglial activation from pro-inflammatory to beneficial [53,54].

As expected, the OGD procedure disturbed the organization of the neuronal cytoskeleton, as reflected by changes in the expression of MAP-2. Our observations are in line with data from other investigators showing a negative effect of ischaemia on the expression of MAP-2 protein [55]. Furthermore, a decrease in MAP-2 levels immediately after MCAo was demonstrated in a mouse model of cerebral ischaemia. Nevertheless, this trend was reversed 7 days after MCAo. At this time point, an increase in the expression of MAP-2 was present, suggesting activation of endogenous repair mechanisms [56].

Growing evidence indicates that dying or damaged cells after ischemia exhibit both nuclear and cytoskeletal changes. Moreover, cytoskeletal protein degradation has been suggested to be a crucial factor in investigating neuronal damage after ischemia. Interestingly, the differences in cell death-related processes such as DNA degradation and cytoskeletal breakdown probably reflect different degrees of ischaemic insults; e.g., mild ischemia such as 5-min bilateral CCA occlusion caused DNA cleavage followed by loss of MAP-2 immunoreactivity, whereas ischemia such as 30-min unilateral CCA occlusion or complete ischemia with decapitation resulted in loss of MAP-2 immunoreactivity faster than nuclear changes in the hippocampus [57]. Nevertheless, although MAP-2 was proposed as a marker of cytoskeletal breakdown in some ischaemia models [58,59], less attention has been given to the cytoskeletal changes induced by the OGD procedure, especially in the ex vivo model.

In this context, the second main finding in the present research is demonstrating the disturbed organization of the microtubular cytoskeleton represented by the depolymerization of microtubules and aggregation of tubulin in the cell body after the OGD procedure (see Figure 6). To date, only limited observations have indicated that cerebral ischaemia can lead to the depolymerization of microtubules in the MCAo model. However, another element of the cytoskeleton, the light subunit of neurofilament (NF-L), namely, significantly increases fluorescence intensity for NF-L in the form of degraded protein fragments [60]. It must be emphasized that the microtubular network is crucial for trafficking cargo to neuronal synapses [61]. In our research, a high susceptibility of the cytoskeleton to hypoxia, including the rearrangement of actin into “spongy structures”, was found. Similar results were observed in an in vivo stroke model as reflected by actin depolymerization and its subsequent aggregation [62]. Previously published results showed that the biomechanical properties in neuroblastoma SH-SY5Y cells subjected to OGD were associated with the remodeling of actin filaments, as well as cofilin-based regulation and impaired metabolic activity of cells, showing the importance of nanomechanics in research on ischaemia-related pathological processes such as stroke [63].

Modulation of mechano-coupled proteins as a therapeutic approach has been recently underlined for various nervous system diseases, including stroke, because physical factors play a significant role in the functioning and activation of brain cells [64]. Some drugs acting on the cytoskeleton have already been applied in cancer treatment since the inhibition of microtubule functions during mitosis leads to apoptosis induction in rapidly dividing cells [65]. Among others, the use of the ROCK kinase inhibitor Y27632 partially limited the depolymerization of actin fibers and the formation of aggregates as well as reduced cell death after ischaemia. Limited neuronal mortality was also observed after the use of the actin polymerization inhibitor latrunculin [66]. Finally, the Ras homolog (Rho)-associated kinase (ROCK) inhibitor fasudil is used in clinical practice in China and Japan for the treatment of stroke. Its action relies on decreasing inflammation due to its impact on T cells, B cells, and microglia but also on promoting nervous tissue regeneration due to the activation of stem cells [67]. In our study, we showed a significant decrease in brain-specific macrophage-derived cells count after OGD. Recently, the research group of Atcha et al. (2021) showed that the response to substrate stiffness by macrophages is modulated by the mechanosensitive transmembrane channel Piezo1, whose expression is elevated on stiff substrates. Consequently, the Piezo1 agonist compound Yoda1 leads to an increase in the expression of the inflammatory marker Nos2 in macrophages growing on stiff substrates, while the use of Yoda1 at high concentrations leads to an increase in the expression of Nos2 in macrophages grown not only on stiff (40 kPa) but also soft (1 kPa) substrates [68]. Piezo1 seems to be a promising target for multiple neurological diseases, especially due to the existence of its modulators (Jedi1, Yoda1, GsMTx-4). In this context, a focus on the cytoskeleton and mechanical cues seems to be one of the future branches of stroke research [69]. These results reveal the importance of the cytoskeleton as well as related protein disturbances in the pathology of ischaemic brain damage, especially as these changes have broader significance by influencing the biomechanical properties of brain cells.

Therefore, in the next stage of our research, using AFM, we analyzed the biomechanical properties of OHCs subjected to OGD. We demonstrated for the first time the significant increase in OHCs stiffness induced by the OGD procedure. To date, data on biomechanical measurements of the brain are sparse. Among them, the analysis in the hippocampus, including the layer of granular cells (GCL) and the sub-granular zone (SGZ), revealed that the stiffness of this brain area increases with aging, which is probably associated with a decrease in the expression of chondroitin sulfate proteoglycans (CSPGs) and consequently in changes in the mechanical properties of the brain tissue in elderly animals [70]. Stiffening of nervous tissue was also determined using AFM after CO_2_-induced brain acidosis in rat brain slices [71]. Importantly, tissue stiffening is a marker of pathological changes in other diseases, including cancer [72] or cardiovascular diseases [73].

Although the mechanical properties of cells and tissue have been the focus of some studies, the role of biophysical forces in the immunological response is less recognized. Recently, it has been shown that the functional activation of cells of the immune system and intercellular mechanical interactions are closely related. Culture of dendritic cells on stiffer (12 kPa) substrates decreased CLR (C-type lectin receptor) expression, CLR-mediated antigen internalization, β2 integrin expression, and CCL21-mediated chemotaxis [74]. To date, the macrophage activation profile has been demonstrated to depend on the mechanical properties of the substrate. Increased expression of IL-1β upon LPS-induced activation was also reported for murine macrophages cultured on stiffer (840 kPa vs. 130 kPa) PEG-RGD substrates [75]. In fact, macrophages growing on stiffer substrates (280 kPa) exhibit higher iNOS expression upon interferon γ and lipopolysaccharide (LPS) stimulation than macrophages growing on soft substrates (20 and 40 kPa). Moreover, IFN γ/LPS-induced NFκB and STAT6 intracellular pathway activation is increased on stiff (280 kPa) substrates.

Consequently, the biomechanical properties of brain cells and tissues are thought to be affected partly by the mechanics of the extracellular matrix (ECM), which undergoes remodeling during inflammation. Several factors may impact the ECM, including cytoskeletal malfunction. However, little is known about the molecular mechanisms influencing the mechanical properties of microglia in ischaemia. Therefore, it is suggested that the changes in OHCs stiffness induced by the OGD procedure, including microglial cells (our unpublished data), may significantly affect the phenotype of these cells and consequently lead to an increase in pro-inflammatory activation as well as secreted cytokines and chemokines, which we demonstrated in the present study. This hypothesis seems to be in line with the observation that the LPS-induced inflammatory activation is substrate stiffness-dependent, with an increased elastic modulus resulting in increased macrophage activation. The secretion of pro-inflammatory cytokines (TNF-α, IL-1β, IL-6) gradually increased with increasing substrate stiffness in the range of 0.3–230 kPa [76]. Due to mechano-sensing, lymphocytes respond to their mechanical environment in a stiffness-dependent matter. Nevertheless, their response depends on the nature of the receptor engaged in sensing the stiffness of the environment. Adhesion mediated only by TCR results in a biphasic stiffness response, while during adhesion mediated by lymphocyte function-associated antigen 1, integrin cell spreading increases monotonically with increasing substrate stiffness [77]. On the other hand, not only do immune cells feel the physical properties of their surroundings, but they also respond mechanically to extracellular stimuli. Activation of leukocytes results in their stiffening up to 10-fold and increased viscosity of cells. This trend was shown for T cells, B cells and leukemic PLB-985 cells, which serve as a model of the neutrophils [78]. All these data underline the crucial role of the mechanical properties of tissues on the profile of the inflammatory response. We are aware that our studies have some limitations. Therefore, to overcome some of them and find a correlation among various parameters obtained in our study, we calculated Pearsons’ and Spearman’s rank correlation coefficients. In OGD-treated samples, among others, linear correlations were found between tissue stiffness and LDH level (Pearson’s coefficient = 0.30782) and tissue stiffness, IBA1 positive branched cells spread area (Pearson’s coefficient = −0.981) and pro-inflammatory CCL3 and actin mean density (Pearson’s coefficient = −0.98955). Based on these correlative analyses, it can be suggested that our research hypothesis indicating the relationship between cytoskeleton changes and pro-inflammatory response induced by OGD in OHCs seems to be positively verified.

Nevertheless, a direct mechanistic relationship required further studies. Furthermore, the molecular basis of the OGD effects on cellular biomechanical changes awaits further investigation. Next, although OHCs is a well-validated model, it does not fully mimic in vivo brain functions. Finally, the performance of the presented studies was only at one time point after the OGD procedure, considering the complexity of time-dependent changes occurring after ischaemia, including reoxygenation, can also be crucial in the interpretation of the presented data. Nonetheless, our current data constitute a thorough basis for further research and provide a rationale for integrating biomechanical and biochemical methods in studying the pathomechanism of stroke-related brain damage.

## 5. Conclusions

Based on the results of the current research, it can be proposed that OGD-evoked changes in neuronal and microglial cell death/viability, as well as dysfunction in the organization of the microtubular and actin cytoskeleton, are reflected in the mechanical properties leading to enhanced stiffness of the hippocampus. Simultaneously, an increase in the level of pro-inflammatory factors, namely, cytokine (Il-1β, IL-18, TNF-α) and chemokine (CCL3, CCL5, CXCL10) release, as well as nitric oxide production provide interesting evidence that there is a potential correlation between biomechanical factors and the inflammatory response. Our observations point out the interesting direction of proof-of-concept studies, and their follow-up may establish new targets for brain ischaemia therapy.

## Figures and Tables

**Table 1:** Antibodies and dyes used for immunofluorescence staining of OHCs.

Antibody/Dye	Dilution	Company
Anti-MAP-2	1:300	Abcam, Cambridge, UK
Anti-IBA1	1:200	Abcam, Cambridge, UK
Anti-beta-tubulin conjugated with Cy3	1:150	Sigma-Aldrich, Saint Louis, MI, USA
Phalloidin conjugated with AlexaFluor488	1:200	Invitrogen, Waltham, MA, USA

**Table 2:** Secondary antibodies used for immunofluorescence staining of OHCs.

Antibody/Dye	Dilution	Company
Donkey anti-goat conjugated with Alexa Fluor 555	1:300	Abcam, Cambridge, UK
Goat anti-rabbit conjugated with Alexa Fluor 647	1:200	Abcam, Cambridge, UK

## Data Availability

All data supporting the conclusions of this manuscript are provided in the text, figures, and tables.
